# Dying from cardiac tamponade

**DOI:** 10.1186/1749-7922-2-22

**Published:** 2007-09-06

**Authors:** Aravind Swaminathan, Karikalan Kandaswamy, Manish Powari, Joseph Mathew

**Affiliations:** 1 Department of Histopathology, Royal Cornwall Hospital, Truro, UK. TR1 3 LJ; 2 Department of Cardiology, Royal Cornwall Hospital, Truro, UK. TR1 3 LJ

## Abstract

**Background:**

To determine the causes of cardiac tamponade (CT), focussing especially on haemopericardium (HP), as a terminal mode of death, within a 430,000 rural English population.

**Methods:**

Our hospital mortuary register and, all postmortem reports between 1995 and 2004 inclusive, were interrogated for patients dying of CT or HP. The causes of CT/HP and selected morphological characteristics were then determined.

**Results:**

14,368 postmortems were performed in this period: of these, 461 patients died of CT. Three cases were due to non-haemorrhagic pericardial effusion. HP accounted for the remaining 458 cases of which, five were post-traumatic, 311 followed rupture of an acute myocardial infarction (RAMI), 138 after intra-pericardial rupture of dissecting ascending aortic aneurysms (RD3A) and four were due to miscellaneous causes.

HP was more commonly due to RAMI. Men tended to die from RAMI or RD3A earlier than women. RAMI or RD3A were commoner in men <70 yrs, but more frequent in women after this.

Two thirds of RAMI were associated with coronary artery thrombosis. Anterior free wall rupture was commonest overall, and in women, but posterior free wall rupture was commoner in men.

The volume of intrapericardial blood in RAMI (mean = 440 ml) and RD3A (mean = 498 ml) varied between 150 and 1000 ml: intrapericardial blood volume was greater in men than in women dying from either RAMI or RD3A.

**Conclusion:**

At postmortem, CT is most often related to HP, attributable to either RAMI or intrapericardial RD3A. Post-traumatic and other causes of CT are infrequent.

## Background

The pathophysiology of cardiac tamponade (CT), as a cause of death, is related to an increase in intrapericardial fluid pressure that exceeds atrial venous pressures, thereby impeding venous return to the heart[1]. Rapidly evolving HP (200 to 300 ml) is more likely to cause death from CT than slowly evolving pericardial fluid accumulation (500 to 2000 ml), the latter allowing for accommodation of greater volumes due to gradual distension of the pericardial sac[1]. The normal volume of pericardial fluid (30 to 50 ml) reflects a balance between production and reabsorption[1].

The causes of CT include active or passive pericardial effusion and haemopericardium (HP) consequent on trauma, iatrogenic intervention or, either rupture of an acute myocardial infarction (RAMI) or intrapericardial rupture of a dissecting ascending aortic aneurysm (RD3A)[1,2]. HP commonly follows RAMI, RD3A or trauma [1-3]; it has also been described in association with malignancy[3,4], chemotherapy[5], homicide[6], pacing wire[7] or central venous catheter[8,9] insertion, endocardial biopsy[10], open heart surgery[11], interventional coronary artery procedures (atheroablative, angioplastic or stenting)[12], exercise stress test[13], electroconvulsive therapy[14], coronary artery vasculitis[15] or dissecting aneurysm[16], myocardial abscess[17], infective endocarditis[3] and, during amniocentesis[18] or in the prenatal period[19]. CT been described in association with pneumopericardium [20] and as a complication of intrapericardial drain insertion[3]. HP has been described as a complication of 5% to 10% of patients with AMI [21][22].

Post-AMI myocardial rupture includes ventricular free wall rupture, ventricular septal rupture or papillary muscle rupture [1,3]. Risk factors for this include age >60 yrs, female gender, pre-existing hypertension and lack of left ventricular wall hypertrophy[1].

The lateral wall, at mid-ventricular level, is said to be the most common site for post-infarction free-wall rupture[1]. However, the lateral and inferior aspects of the left ventricle have been reported as equally susceptible to post-infarctive rupture [21][23].

We have reviewed all cases of patients dying of cardiac tamponade in our hospital over a ten-year period to determine the causes of cardiac tamponade and highlighted some associated morphological features.

## Methods

Our department performs postmortems at the request of Her Majesty (HM) Coroner, in the County of Cornwall, that has a population of ~430,000. Almost all deaths in this catchment area, falling within the jurisdiction of HM Coroner, are sent to our mortuary for a postmortem. These deaths are principally those within the community, or hospital, that fall within the purview of HM Coroner [24]: for the most part, these postmortems are performed because there is no firmly established cause of death.

The Mortuary Death Register of the Department Histopathology, Royal Cornwall Hospital, was reviewed for causes of death listed as either "haemopericardium" or "cardiac tamponade" between 1995 and 2004 (inclusive). These results were compared to a search of our textual postmortem report database using "moper" (a unique string of letters in the word "haemopericardium") and "tamponade" as separate search criteria within the text of the reports; the results of these were amalgamated and duplicates discarded.

All postmortem reports were retrieved and the cause of death confirmed as being attributable to HP or CT; the following parameters were then confirmed and retrieved from each postmortem:

1. The gender and age of patients

2. The causes of the CT or HP

3. If HP due to myocardial infarction, the

a. Presence of coronary artery thrombosis

b. Site of myocardial rupture and

4. Volume of intrapericardial haemorrhage

## Results

**Figure 1:**
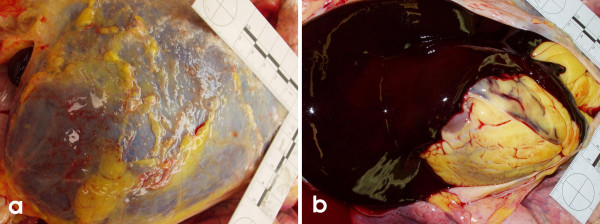
This image depicts a) the characteristic bluish black pericardial distension, observed at postmortem, most often b) containing an admixture of clotted and frank blood.

**Figure 2:**
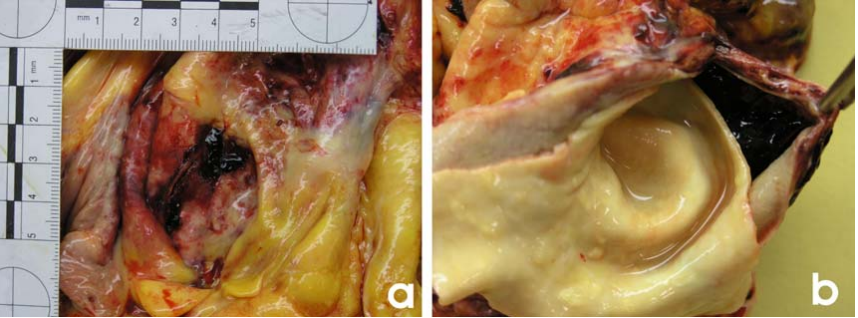
This image shows the a) intrapericardial site of rupture of a dissecting aneurysm of the ascending aorta, close to its origin and b) the presence of blood within such a dissection.

14,368 postmortems were performed at the Royal Cornwall Hospital in the ten years under review. There were 461 cases of CT (3.2% of all postmortems), of which there were three cases of non-haemorrhagic effusion (passive = 2; infective = 1), five cases of post-traumatic HP and four miscellaneous causes (Table 1); these were excluded from subsequent analysis. Of the rest (n = 449; 98%), CT was due HP (Figure 1) secondary to RAMI (n = 311; 69%) or RD3A (n = 138; 31%) (Figure 2).

**Table 1:** Other causes of haemopericardium.

Cause of HP	Sex	Age
*Left ventricular rupture following RTA	male	19
*Partial avulsion of right pulmonary vein	male	33
*Avulsion crush injury to chest	male	35
*Stab injury	female	83
*Perforation of right ventricle	male	84
Angiosarcoma of pericardium	female	38
Metastatic carcinoma from lung	male	69
Warfarin therapy	female	77
Malignant pericarditis (cause unknown)	male	78

This table defines the causes of HP, other than RAMI or RD3A, causing cardiac tamponade. There were * 5 cases of post-traumatic HP. Four patients died of HP from other causes.

**Figure 3:**
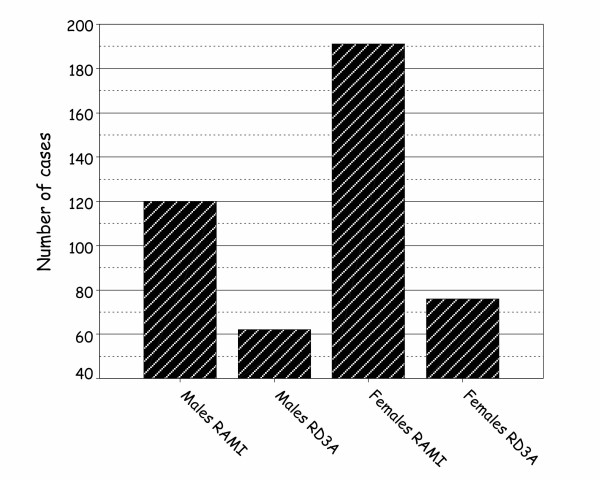
Cumulatively, more women die from RAMI or RD3A than men do.

Death due to HP, as a consequence of either RAMI or RD3A, is commoner in women than men (Figure 3). RAMI is a more common cause of HP than RD3A in both men and women.

**Figure 4:**
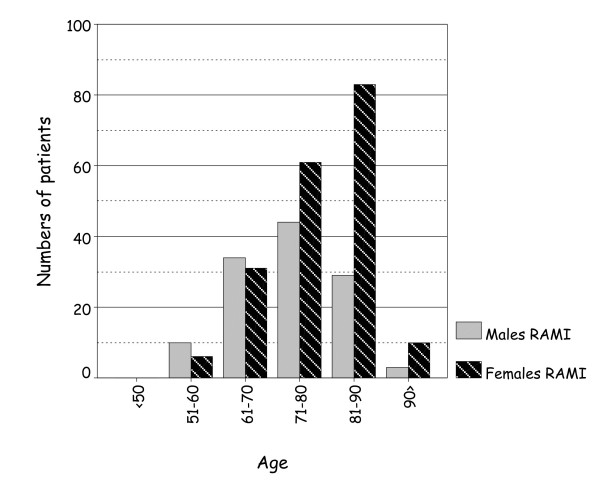
Men die from RAMI more frequently, than women do, before the age 70 years than after it.

**Figure 5:**
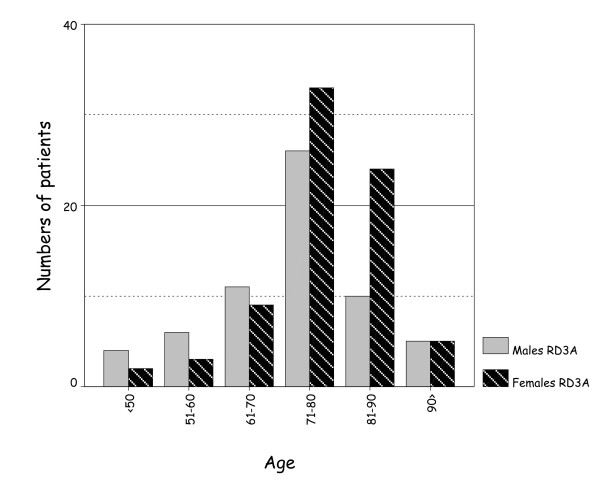
More women, than men, die from RD3A after the age of 70 years, than before it.

The age range of non-traumatic HP in men (33 to 99 years) and women (21 to 99 years) are similar. The mean age of men dying of HP/CT as a result of RAMI (73.4 ± 8.6) (n = 120) and RD3A (72.5 ± 13.0) (n = 62) is less than those of women dying of HP/CT from RAMI (78.7 ± 9.0) (n = 191) and RD3A (76.6 ± 11.1) (n = 76). The incidence of RAMI and RD3A in each age group is shown in Figures 4 &5; death from HP due to either cause is commoner in men than female before 70 yr but reverses after this and is commoner in women.

### RAMI

RAMI was responsible for most causes of death (69%; n = 311) due to HP, with an overall male (n = 120; 39%) to female ratio (n = 191; 61%) of 1:1.6.

Coronary artery thrombosis (CAT) was recognised in 210 postmortems (67%: 82 M, 128 F); CAT was not identified in the remainder (n = 101: 38 M, 63 F). There were no significant differences in the ages of men or women with CAT as a cause of RAMI.

The site of ventricular rupture was defined in all postmortem reports (table 2), with the anterior wall (33.8%) being the most common site of rupture. Cumulative figures show anterior (42.8%) and posterior ventricular wall rupture (37.3%) being more common than apical (6%) or lateral (22.5%) ventricular wall rupture. These figures are probably skewed by the larger numbers of females in the RAMI group.

**Table 2:** Site of rupture of acute myocardial infarction.

Site of rupture	Men	Women	Total	Cumulative figures, by site		
Apex	6 (1.9)	13 (4.2)	19 (6.1)	Apex 6%		
Anterior	33 (10.6)	72 (23.2)	105 (33.8)	Anterior n = 133 (42.8)		
Antero-septal	5 (1.6)	9 (2.9)	14 (4.5)			
Antero-lateral	4 (1.3)	10 (3.2)	14 (4.5)		Lateral n = 70 (22.5)	
Lateral	23 (7.4)	18 (5.8)	41 (13.2)			
Postero-lateral	8 (2.6)	7 (2.3)	15 (4.8)			Posterior n = 116 (37.3)
Posterior	38 (12.2)	60 (19.3)	98 (31.5)			
Postero-septal	1 (0.3)	2 (0.6)	3 (1.0)			
Total	120 (38.6)	191 (61.4)	311 (100.0)			

This table shows the documented sites of RAMI's in men and women dying from HP/CT. Figures in () define the % occurrences of each type of ventricular free wall rupture to the total population of patients with RAMI. Cumulative figures represent the total of each of anterior, lateral or posterior free wall rupture as described, alone or in combination.

Men were more likely to have posterior (12.2%) than anterior (10.6%) or lateral (7.4%) ventricular wall rupture; women, by contrast, had anterior (23.2%) ventricular wall rupture more commonly than posterior (19.3%) or lateral (5.8%) wall rupture.

### RD3A, HP and CT

Patients dying from intrapericardial RD3A formed a smaller but significant group (30.5%; n = 138) of patients dying from HP. This mode of death was slightly more frequent in women (n = 76; 55%) than in men (n = 62; 45%) (Figure 3).

### Intrapericardial blood volume

**Figure 6:**
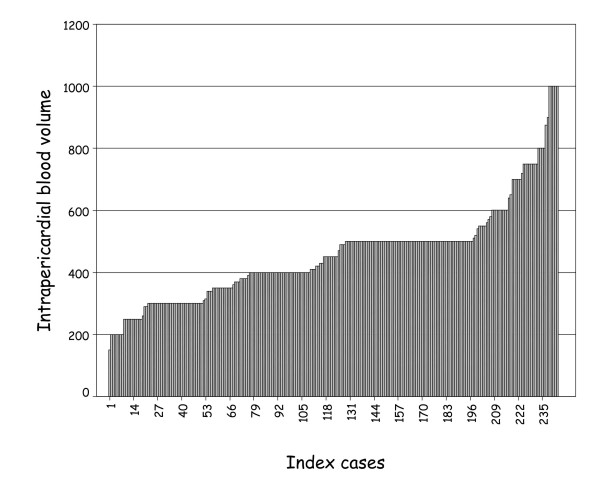
This shows the volume of intrapericardial blood in each of 246 (index) cases where this was recorded.

The volume of blood in the pericardial space was recorded in 246 (54%) instances (99 M, 147 F); this ranged between 150 ml and 1000 ml of blood in RAMI or RD3A (figure 6).

In addition, 800 ml of blood was seen in association with pericardial angiosarcoma, 750 ml in association with metastatic lung carcinoma and 1650 ml in association with malignant pericarditis. The volume of blood in association with Warfarin therapy was not recorded.

A difference in intrapericardial blood volume was noted between men and women whether as a consequence of either RAMI (M = 473 ml, F = 418 ml) or RD3A (M = 514 ml, F = 474 ml).

Death was associated with a slightly smaller volume of HP in-patients dying as a consequence of RAMI (mean = 440 ml) when compared to RD3A (mean = 498 ml).

## Conclusion

There is little evidence in the literature reflecting on the causes or demographics of HP in routine postmortem practice. With the exception of individual case reports [4][6][7][9][10][11][12][13][14][15][16][17][18][19][20], most of the evidence available in standard textbooks is non-referenced.

Only 3.2% of all our deaths, over a 10-year period, were attributable to CT. In our series RAMI and RD3A were the principal causes of CT, causing death as a consequence of HP. Pericardial effusion, post-traumatic CT and miscellaneous causes of CT together formed only a small group of CT-related deaths.

Although the lateral wall at mid-ventricular level [1][21][23] or the inferior wall [21]are said to be the most common sites for post-infarction free-wall rupture [1][21][23], in our series anterior left ventricular wall rupture was more common than rupture at other ventricular wall sites. This might be a reflection of the greater numbers of women in our study, anterior free wall rupture being more common in women and posterior free wall rupture in men.

By contrast to traditional risk factors of female gender and age >60 years[1], men appear to die from CT due either RAMI or RD3A earlier than women. Indeed, death from either of these causes is commoner in men than women before, but not after, 70 years of age. This probably reflects the effects of cardiogenic risks in women evolving in the postmenopausal period and of men dying earlier from other causes, including AMI without myocardial rupture.

Traditionally, acute cardiac tamponade is associated with between 200 ml and 300 ml of sudden accumulation of intrapericardial fluid[1] or, in chronic slowly evolving accumulation, of volumes between 1000 and 2000 ml[1,3]. This contrasts our experience with volumes of between 440 ml and 500 ml in association with CT due to acute pericardial accumulation of blood, in women and men respectively. In cases where HP volumes were larger than this, we speculate that this was associated with a slow leak, with or without a terminal phase of catastrophic rapid haemorrhage. We also demonstrated that slightly less intrapericardial blood volume is associated with RAMI than RD3A in fatal HP.

In conclusion we have defined the aetiology of CT in a postmortem population in rural South-West England and have described some of the salient morphological features associated with CT.

## Abbreviations

CT – Cardiac Tamponade

HP – Haemopericardium

AMI – Acute Myocardial Infarction

RAMI – Ruptured Acute Myocardial Infarction

D3A – Dissecting Ascending Aortic Aneurysm

RD3A – Rupture Dissecting Ascending Aortic Aneurysm

PM – Postmortem

## Competing interests

The author(s) declare that they have no competing interests.
